# Similarities and differences of bone marrow and peripheral blood samples from acute myeloid leukemia patients in terms of cellular heterogeneity and ex‐vivo drug sensitivity

**DOI:** 10.1002/jha2.961

**Published:** 2024-06-17

**Authors:** Gulser Caliskan, Yudi Pawitan, Trung Nghia Vu

**Affiliations:** ^1^ Department of Medical Epidemiology and Biostatistics Karolinska Institutet Stockholm Sweden

**Keywords:** AML, bone marrow, cellular heterogeneity, drug sensitivity, peripheral blood

## Abstract

**Background:**

Bone marrow (BM) evaluation is the de facto standard for diagnosis, molecular analysis, risk stratification, and therapy response assessment in acute myeloid leukemia (AML), but in patients with a high number of circulating blast cells, the peripheral blood (PB) sample could provide similar information as BM. However, there is no large‐scale molecular study comparing the two specimens in terms of their gene expression profiles, cellular heterogeneities, and ex‐vivo drug sensitivity.

**Methodology:**

We used (i) the BEAT‐AML cohort each with detailed molecular data; (ii) cell‐type deconvolution to estimate leukemic and immune cell proportions between specimen types; (iii) differential expression (DE) and drug‐cell type association analysis; and (iv) logistic regression models to assess the association between induction therapy response, cell‐type composition and first‐line drug treatment.

**Results:**

Results: We identified 207 patients having BM and 116 patients having PB samples. There was a total of 1271 DE genes (false discovery rate < 0.05) between BM and PB; the top enriched pathways in terms of DE genes belong to the immune system pathways. Aggregated ex‐vivo drug response profiles from the two specimens were largely similar, as were the cellular components, except for the GMP‐like cell type (17% in BM vs. 5% in PB, *p*‐value = 2 × 10^−7^). Among the specimen‐specific results, the GMP‐like subtype was associated with multiple drug resistance in BM and the ProMono‐like subtype in PB. Several cell types were associated with the response to induction therapy, but the impact of specimen type on the interaction of cell type and cytarabine‐associated induction therapy was not statistically significant for most cell types.

**Results:**

Conclusions: Even though there are molecular and cellular differences between BM and PB samples, they show many similarities in ex‐vivo drug response profiles, indicating the clinical utility of the substantially less‐invasive PB samples.

## 1 INTRODUCTION

Acute myeloid leukemia (AML) is a blood cancer characterized by accumulated somatic mutations and aggressive clonal proliferation in hematopoietic stem cells (HSC) [1]. It is the most common form of acute leukemia with a recent estimated global incidence of 1.85 per 100,000 person‐years in males and 1.37 per 100,000 person‐years in females [2]. AML is usually diagnosed by the percentages of immature blast cells (>20%–30%) in either peripheral blood (PB) or bone marrow (BM); the specimen type is usually decided by the treating physician [3, 4]. BM biopsy at diagnosis is considered the gold standard, but it is an invasive procedure. A PB sample is much more easily accessible and, if there are enough circulating blast cells, it could be sufficient for clinical purposes. Both specimen types are widely used in AML studies because there is evidence of a strong correlation between PB and BM samples in terms of flow cytometric and cytogenetic analyses [5], mutation analysis [4, 6], and proteomic patterns [7]. BM and PB AML samples are from different tissues, so they can be expected to have differences in molecular profile. However, current studies do not fully describe the similarities and differences of the specimen types in terms of their gene‐expression profiles, cellular heterogeneities, and how they correlate to ex‐vivo drug sensitivity.

The relationship between cellular heterogeneity and drug response has gained increased attention in recent AML research. For example, using 14 AML cell type‐specific genes [8], Zeng et al. investigated the leukemia cell hierarchy of more than 1000 AML patients using deconvolution of bulk RNA‐sequencing (RNA‐seq) data [9]. They discovered different associations between the cell‐type proportions with drug sensitivities, AML genomics features such as mutations and cytogenetic events, and patient prognosis [9]. Similarly, Bottomly et al. showed the broad influence of AML cell maturation state on drug sensitivity, and sometimes conditional on other factors like mutational/cytogenetic events [10]. Investigating the impact of mutational clones on the drug response of AML, Benard et al. identified several mutations that can predict drug sensitivity based on their subclonal abundance [11]. In a 2022 study, Karakaslar et al. utilized 1350 bulk RNA‐seq samples from five AML cohorts for the deconvolution of immune cell types to recapitulate the entire FAB landscape (M0–M7) and predict responses of patients to venetoclax [12]. Finally, Beneyto‐Calabuig et al. showed that AML patients with the most immature leukemic progenitors had blast persistence or died during the first induction therapy, but patients with lympho‐myeloid primed progenitors (LMPP)‐like leukemic progenitors went to complete remission [13]. Overall, these studies have demonstrated the association of cellular heterogeneity with drug sensitivity and clinical response in AML. However, to what extent these results are influenced by specimen types has not been thoroughly investigated yet.

In this study, to compare the utility of BM and PB samples from AML patients, we investigated their molecular and cellular differences in terms of transcriptomics and cell‐type profiles via deconvolution of 443 samples from the BEAT‐AML cohort [10, 14]. We then explored the association between specimen type and ex‐vivo drug response at whole tissue and cell‐type levels. We further investigated the impact of specimen type on the analysis of the relation between cell‐type composition and response to induction therapy.

## 2 MATERIAL AND METHODS

### 2.1 BEAT‐AML cohort

This cohort [10, 14] has been designed and implemented in a precision medicine trial in AML. The dataset consisted of 805 AML patients (942 specimens) and included genomic and transcriptomic profiles, clinical annotations, and ex‐vivo drug response data. We collected 443 samples with the specimen type of either BM aspirate or PB which are collected at the initial diagnosis. Because of the small sample size, the samples with the specimen type of Leukapheresis (*n* = 13) were excluded. For gene expression data, we download the raw gene expression count (hg38) of the BEAT‐AML cohort from https://portal.gdc.cancer.gov. This data matrix contains 56,167 genes from 443 patients. Drug response data of these patients are obtained from the original study [10], including a total of 34,797 records from 338 patients across 165 drugs. The drug sensitivities are measured by area under the dose‐response curve (AUC), IC50, and EC50. The AUC is a robust metric used in the analysis of the original study in which a lower AUC indicates a better drug response.

### 2.2 Cell‐type deconvolution

CIBERSORTx [15] is applied to estimate cell‐type compositions from the raw gene expression count of the AML patients. We selected CIBERSORTx for the analysis as it is the top‐performing method in the context of AML [9]. This study focuses on the cell proportions of seven leukemic cell types (LSPC‐quiescent, LSPC‐primed, LSPC‐cycle, GMP‐like, Mono‐like, ProMono‐like, and cDC‐like) and seven non‐leukemic immune cell‐types (Naïve T, cytotoxic T lymphocytes [CTL], natural killer [NK], B, plasma, monocyte, and cDC) which are described in details from the recent AML study [9]. We also follow the implementation approach for the deconvolution described in that study. Specifically, the gene expression of the single cells [9] of those cell types is used as input for the generation of the signature matrix with CIBERSORTx [15]. Deconvolution is performed on raw bulk RNA‐seq data using S‐mode batch correction and Absolute mode. Other settings are used with the default values.

The Wilcoxon rank sum test is performed to evaluate differences in cell type proportions between specimen types. The p‐values are extracted and the false discovery rate (FDR) is calculated using the Benjamini & Hochberg method to account for multiple testing [16]. Differential expression (DE) analysis is performed on raw gene expression count using DESeq2 [17].

### 2.3 Association of cell‐type composition with drug sensitivity and induction therapy

Of 443 patients, 338 (*n* = 209 of BM and *n* = 129 of PB) together with the ex‐vivo drug sensitivity (AUC) data of n = 155 drugs are kept for the analysis. For individual pairs of a drug and a cell type, the Spearman correlation coefficient between the AUCs of the drug and the proportions of the cell type of the AML patients is calculated. Then, we define a cell type as sensitive to a drug, if the correlation coefficient is negative and the test is statistically significant (*p* < 0.05); vice versa, a cell type is defined as resistant to a drug if the correlation is positive and statistically significant.

A logistic regression model is used to investigate the association between the response to induction therapy of AML patients, cell‐type composition, and a drug used in the first‐line treatment. We perform three separate logistic regression models for the patients with specimen type of BM (*n* = 207), PB (*n* = 116), and all patients (group All, *n* = 323). For each of the models, the outcome is the response to induction therapy (complete response/refractory) while the independent variable includes the proportions of all cell types, and the status of using the drug of interest for the first treatment (yes/no). The model is also adjusted for the gender and age of the patients.

All statistical analyses are performed by using R 4.3.1.

## 3 RESULTS

### 3.1 Differential gene expression profiles between BM and PB

**FIGURE 1:**
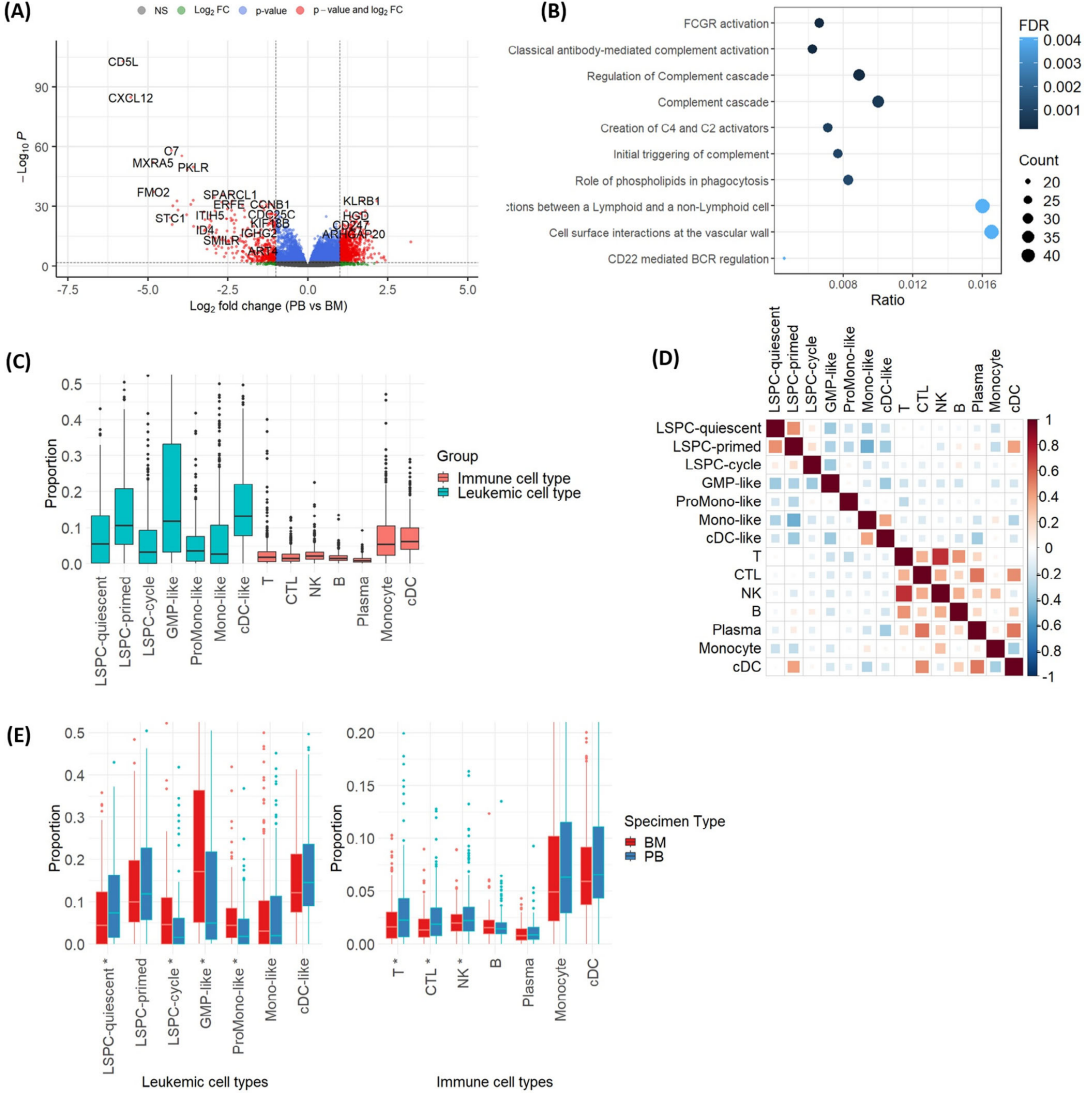
Differential gene expression and cell‐type composition between bone marrow (BM) and peripheral blood (PB). (A) Volcano plot of the differential expression (DE) analysis using DESeq2. The vertical dash lines at −1 and 1, indicate the threshold of log2 fold change for the DE genes. The positive log2 fold change indicates gene expression in PB is higher than in BM and vice versa. The genes above the horizontal line have a false discovery rate (FDR) < 0.05. The grey points are not significant genes (NS); the red points are genes that satisfy both *p*‐value and log2 fold change condition; the blue ones are the remaining. (B) Top 10 significant pathways from the DE genes discovered by DESeq2. Most of them are the pathways from the immune system. (C) Distribution of cell type proportion by leukemic (green) and immune (red) cell types; (D) High correlation of cell type proportions between 14 cell types including seven leukemic cell types (top) and seven immune cell types (bottom); (E) Comparison of cell type proportions between BM and PB in leukemic cell types (the left panel) and seven immune cell types (the right panel). The cell types with * have statistically differential cell type proportions between BM and BP.

We observed large differences in transcriptomics profiles between BM and PB samples. From the DESeq2 analyses, we discovered 1,271 differentially expressed genes (FDR < 0.05) between BM and PB samples, Figure 1A. Top DE genes including *CD5L*, *CXCL12*, *C7*, and others are mostly immune‐related genes, indicating there are differences in immune components between BM and PB samples in the cohort. The pathway enrichment analysis using Reactome [18] on the set of DE genes also confirms this observation. The top 10 significant pathways by FDR, Figure 1B, mostly belong to the immune system pathways. These DE analysis results suggest differences in cell‐type composition between BM and PB samples, which we showed below.

### 3.2 Differential drug sensitivity between BM and PB

We next investigated the differences between BM and PB samples in terms of ex‐vivo drug sensitivities. The ex‐vivo drug experiments in the BEAT‐AML cohort were performed at the tissue level. We include 120 drugs that have at least *n *= 30 patients in each group. The t‐test is applied for the AUCs of both patient groups to identify potential drugs with differential drug sensitivity. The results in Figure S1 show that at the whole tissue level, there is little difference in the AUCs across all the drugs, except perhaps for two drugs (BIRB 796 and BI‐6727), which are not statistically significant when adjusted for multiple testing. The results suggest that an investigation at the cell‐type level is required if we want to see more subtle differences.

### 3.3 Differential cell‐type composition between BM and PB

Figure 1C presents the proportion of the 14 cell types deconvoluted from the bulk RNA‐seq gene expression of the BEAT‐AML cohort. Leukemic cell types generally have higher proportions in comparison with the immune cell type. The dominant leukemic cell types are cDC‐like, GMP‐like, and LSPC‐primed with a median proportion greater than 10%. For the immune cell types, Monocytes and cDC are the top cell types (> 5%). There is a high correlation within immune cell types, similarly for leukemic cell types, blue squares in Figure 1D. However, in leukemic cell types, GMP‐like, ProMono‐like, Mono‐like, and cDC‐like tend to have negative correlations (red squares).

Next, we investigate the impact of specimen type on the cell‐type composition. The greatest difference was observed for the proportion of GMP‐like cell type (17% in BM vs. 5% in PB, *p*‐value = 2 × 10**^−7^**; Table S1). Otherwise, we observed small though statistically significant differences in the proportion of LSPC‐quiescent, LSPC‐cycle, and ProMono‐like, Figure 1E and Table S1. For the immune cell types, BM and PB samples are also largely similar, except for small but statistically significantly higher proportions of CTL, NK, and T cells in PB, Figure 1E and Table S1. In the next section, we will investigate the impact of specimen type on the association between cell type composition and drug response.

### 3.4 Association between cell type composition and drug sensitivity by specimen type

**FIGURE 2:**
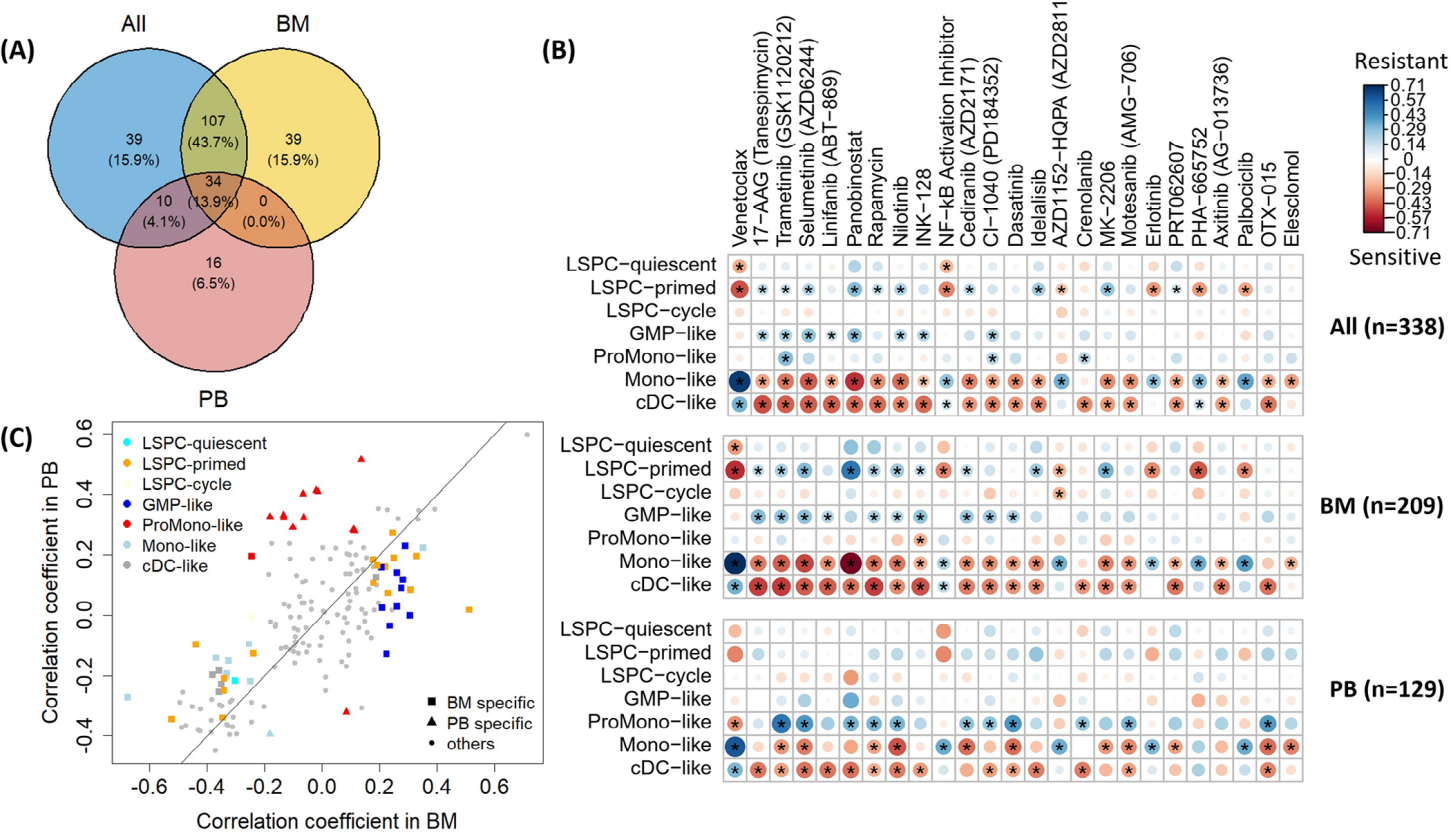
Association between leukemic cell types and drug response. (A) The Venn diagram of the significant correlations (false discovery rate [FDR] < 0.05) between cell‐type proportions and drug response across all acute myeloid leukemia (AML) patients (All groups), patients with BM samples, and patients with peripheral blood (PB) samples; (B). The correlation plot of the top 25 drugs containing the most significant correlations across all patient groups. The order of the drugs (left to right) is based on their increasing minimum *p*‐values across cell types in the All group. The color (red to blue) and size represent the direction (sensitive to resistance) and magnitude of the correlation, respectively. The asterisks indicate the significant correlation (FDR < 0.05); (C) The Spearman correlation coefficients in BM samples versus PB samples.

To investigate the impact of specimen type on the association between cell type composition and drug response, we first calculate the Spearman correlation between proportions of seven leukemic cell types and drug sensitivities of 155 drugs in AML patients. This calculation is also performed across all patients (All groups, *n* = 338) and separately for patients in BM (*n* = 209) and PB (*n* = 129) groups, Figure 2 and Figures S2–S4. A total of 245 cases from 110 out of 155 drugs with significant correlation (FDR < 0.05) between drug and cell type in at least one group, Figure 2A. The Venn diagram shows a high concordance (57.7%) between BM and All groups, which is not surprising since the BM group is much larger than PB. In contrast, PB contains only 18% common cases with the All group.

The correlation results of the top 25 drugs with the most significant correlations across three patient groups are presented in Figure 2B. The resistance of Mono‐like to venetoclax in BM is the most significant correlation (ρ = 0.71, FDR = 7.8 × 10^−23^), and it is also significant for other patient groups. The result is well concordant with the findings from the previous study [9]. However, 16 correlations are significant in PB but not in other groups, and they are mostly found in the ProMono‐like cell type. This cell type is also significantly different in cell‐type proportion between BM and PB as shown in the previous section, Figures 2B‐C. Another differential proportion cell‐type, GMP‐like, together with LSPC‐prime mainly contributes to the case of correlations significant in BM but not in PB, Figure 2B,C. Altogether, the results show that at the cellular level, there are some differences in ex‐vivo drug responses, particularly in relation to the GMP‐like and ProMono‐like cell types.

We also observed similar patterns for the seven immune cell types, Figure S5. Of 155 drugs, 45 have a significant correlation (*n* = 77) with the cell types (FDR < 0.05). Only five cases are significant in PB, and four of them are significant in other patient groups. As with the leukemic cell types, the most significant correlations found in BM are captured in the All group. However, 45.5% of correlations are discovered only in the All group, reflecting the impact of a larger sample size.

### 3.5 Association between cell‐type composition and response to induction therapy

In this section, we investigate the association between cell‐type composition and response to induction therapy. We collected 323 patients with available information on the outcome of the first treatment: *n* = 226 (70%) complete response and *n* = 97 (30%) refractory. Overall, there was no statistically significant difference in the refractory rates (*p*‐value = 0.50) between patients with BM samples (59/207 = 29%) and those with PB samples (38/116 = 33%). This suggests that there was no selection bias in the BM or PB samples in relation to response to induction therapy.

A total of 40 distinct drugs were prescribed for the first treatment across the 323 AML patients, Table S2. Only 55 AML cases (17.03%) received a single drug while the other patients received a combination of 2 or more drugs during their first treatment, Table S3. Due to the limitation of the available ex‐vivo drug data in the BEAT‐AML cohort, we focus on cytarabine which is used in 197 AML patients in the standard chemotherapy, the majority (*n* = 139) are from 7+3 regimen (cytarabine and idarubicin) or (cytarabine and daunorubicin). From the correlation analysis for cytarabine, the LSPC‐primed and cDC‐like cell types are sensitive to the drug, but the Monocyte cell type is resistant in the three groups, Figure S6. However, the significant correlations (FDR < 0.05) were observed mainly in the All group, which is due to its larger sample size.

To explore the association of individual cell types and response to induction therapy, we apply logistic regression using the result of the induction therapy (complete response and refractory) as the outcome, and the proportion of the cell type and the use of cytarabine (used/not used) as the predictors. The model is adjusted for the age and sex of the patients. The results suggested that LSPC‐quiescent, LSPC‐primed, LSPC‐cycle, and GMP‐like cell types are strongly associated with the response to induction therapy (FDR < 0.05), Table S4. Thus, the association with the LSPC‐primed cell type is the only one supported by both the correlation analysis of cell type vs ex‐vivo drug assay and cell type vs induction therapy. There are no predictive signals from the remaining cell types.

When considering the interaction term between the cell type and cytarabine use in the model, only cDC obtains a statistical significance (FDR < 0.05), Table S5. The interaction for cDC is illustrated in Table S6. This 2 × 2 table presents the relation between the cytarabine groups versus the groups of patients with low and high cDC which are divided by the median of cDC proportion. The refractory rate of the cytarabine‐treated patients with low cDC proportion is 0.37, which is 1.4 times higher than the one of the patients with high cDC proportion (0.26), Table S6. The opposite results are observed for patients not using cytarabine, which is 2.5 times greater for the high‐cDC patients. When considering this analysis separately for BM and PB groups, we observe a different pattern in the patients who do not receive cytarabine, Tables S7 and S8. Particularly, for these patients, the refractory rates of the two cDC groups in PB are similar, but in BM this refractory rate is four times greater in the high‐cDC patients compared to the low‐cDC patients.

## 4 DISCUSSION AND CONCLUSION

Both BM and PB samples are often used for the diagnosis and risk stratification of AML patients. However, since they are originally from different resources, we expect some biological differences between them. In this study, we have explored the similarities and differences of the transcriptomics profiles, cell type compositions, and response to therapies between the two specimen types. The cell type compositions are derived by the deconvolution of the gene expression from bulk RNA‐seq of the BEAT‐AML cohort. We then investigate how the differences impact the correlation analysis between cell types and ex‐vivo drug sensitivities. We further explore its influence on the analysis of the relation between cell‐type composition and response to induction therapy.

Even though the comparison between BM and PB samples for AML analysis has been widely investigated, for example, in flow cytometric and cytogenetic analyses [5] or mutation analysis [4, 6], how specimen types affect the analysis of cell type composition has not been extensively investigated. Our study reveals some differences in cell type composition between the two specimen types, which might lead to differential association between the derived cell types with drug sensitivity, including response to induction therapy. The Venn diagrams in Figure 2 and Figure S5 show that some results are specific to either BM or PB. However, the correlations in BM and PB have a similar direction, that is, we do not see evidence of opposite results, for example, of a cell type that is sensitive to a drug in BM but resistant to that drug in PB. Some well‐studied signals such as the resistance of Mono‐like cell types to venetoclax are also consistent both in BM and PB samples.

Intensive induction therapy of the standard frontline AML treatment for the past decades has been 7+3, or 7 days of cytarabine + 3 days of anthracycline‐based cytotoxic chemotherapy such as daunorubicin. However, approximately 40% of AML patients do not respond to the frontline treatment [19]. The differential responses are due to the biological and clinical complexity and heterogeneity such as cell type composition of the disease. Our results support this with strong associations between several cell types and the response to induction therapy. When investigating the impact of specimen type on the interaction of cell type and cytarabine‐associated induction therapy, most cell types do not get statistical significance.

The study's strength is the large number of both BM and PB samples, thus allowing robust statistical comparisons between them, especially for rare cell types. This study also has some limitations. For example, due to data unavailability, the investigation of the impact of specimen types on the first treatment is performed only on cytarabine, a drug that is usually used in combination with other drugs. Thus, this limits the power of the analysis. However, the issue can be improved when more data is released in the future. Even though this study is conducted on the BEAT‐AML cohort, the largest public AML cohort with integration of multiple omics data, drug data, and clinical data, a validation on an independent AML cohort will strengthen the results. Although there are available single‐cell RNA‐sequencing AML data [8], they are all from BM samples, which precludes validation of the differences in cell type composition between specimen types at the single‐cell level.

To conclude, while there are some biological differences such as in global gene expression and cell type proportion between BM and PB, they still have many similarities in ex‐vivo drug response profiles, indicating the clinical utility of the less‐invasive PB samples.

## AUTHOR CONTRIBUTIONS

Gulser Caliskan collected data and performed cell‐type deconvolution. Gulser Caliskan and Trung Nghia Vu performed data analysis and interpretation with input from Yudi Pawitan. Trung Nghia Vu and Yudi Pawitan designed and supervised the study. Gulser Caliskan, Yudi Pawitan, and Trung Nghia Vu contributed to the manuscript writing; and all authors provided final approval of the manuscript.

## CONFLICT OF INTEREST STATEMENT

The authors declare no conflict of interest.

## ETHICS STATEMENT

Not applicable because the study uses public datasets.

## PATIENT CONSENT STATEMENT

The authors have confirmed patient consent statement is not needed for this submission.

## CLINICAL TRIAL REGISTRATION

The authors have confirmed clinical trial registration is not needed for this submission.

## Supporting information

Supporting Information

## Data Availability

The data in this study is based on the BEAT‐AML cohort study with the accession ID at the dbGaP of phs001657.v2.p1. More information about the data is described in the section on MATERIALS AND METHODS.
