# Supplementary material for: Similarities and differences of bone marrow and peripheral blood samples from acute myeloid leukemia patients in terms of cellular heterogeneity and ex‐vivo drug sensitivity
Source: EJHaem. 2024 Jun 17;5(4):721–7. doi: 10.1002/jha2.961 (PMC11327724; doi:10.1002/jha2.961)
Supplement: Supplementary file 1 — Supporting Information [file JHA2-5-721-s001.docx]

**Supplementary documents**

**FIGURES**

**
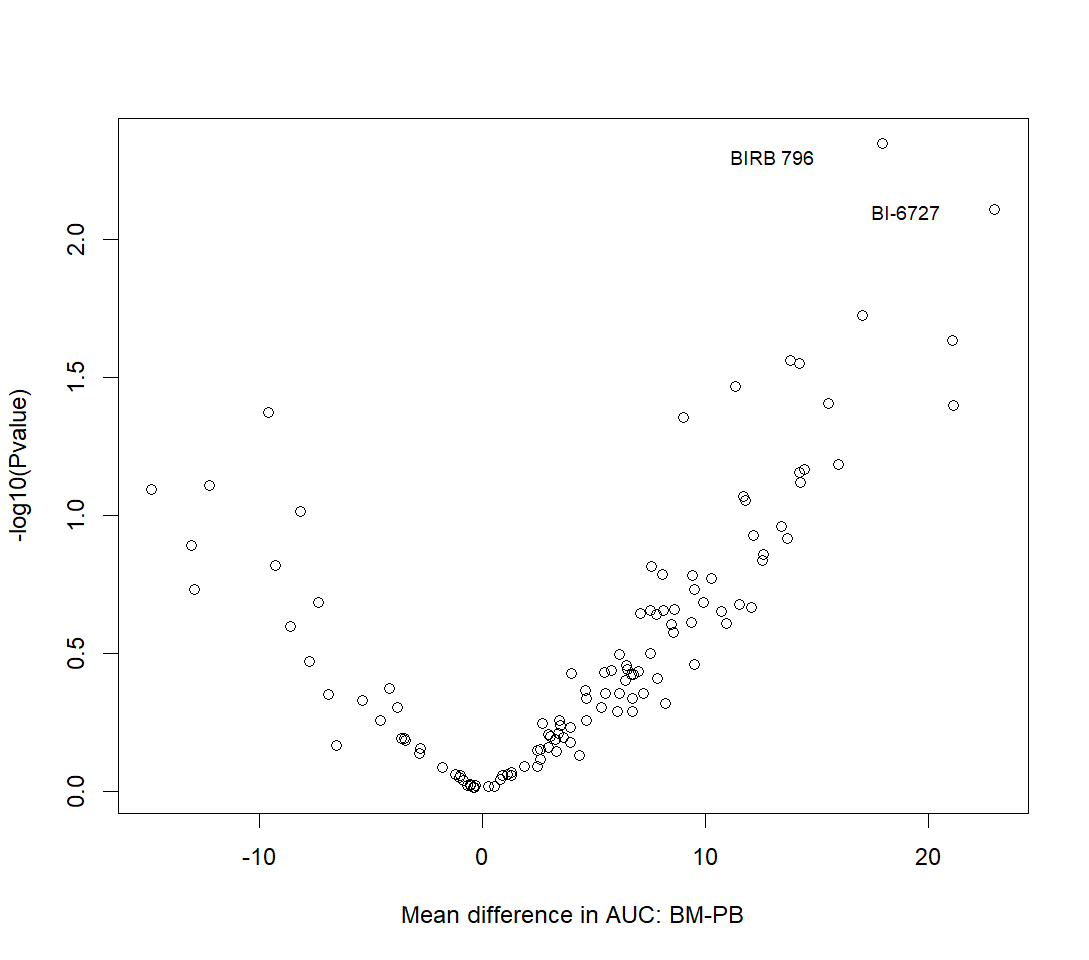
**

**Figure S1. Analysis of differential drug sensitivity between BM and PB**. The x-axis presents the mean difference of AUC between BM and PB. The y-axis presents the -log10(p-value) calculated using t-test. BIRB 796 and BI-6727 are two drugs with p-value < 0.01.


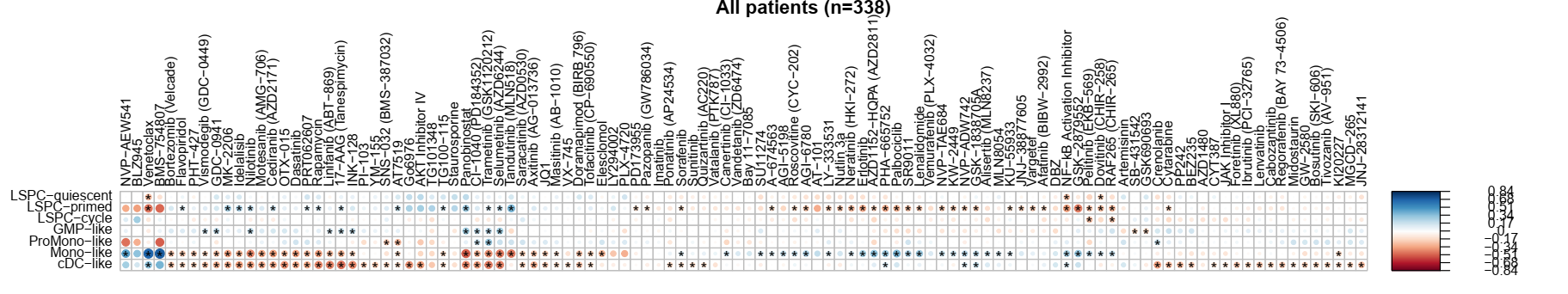


**Figure S2. The correlation plot of 110 drugs containing most significant correlations in group All**. The order of the drugs (left to right) is based on their increasing minimum p-values across cell types in the All group. The color (red to blue) and size represent the direction (sensitive to resistant) and magnitude of the correlation, respectively. The asterisks indicate the significant correlation (FDR < 0.05)


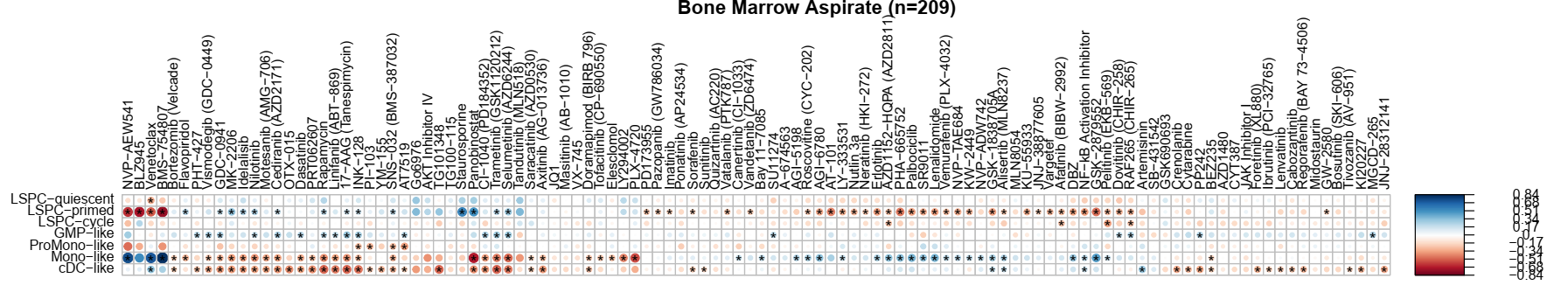
**Figure S3. The correlation plot of 110 drugs containing most significant correlations in group BM**. The annotation is similar to Figure S1.


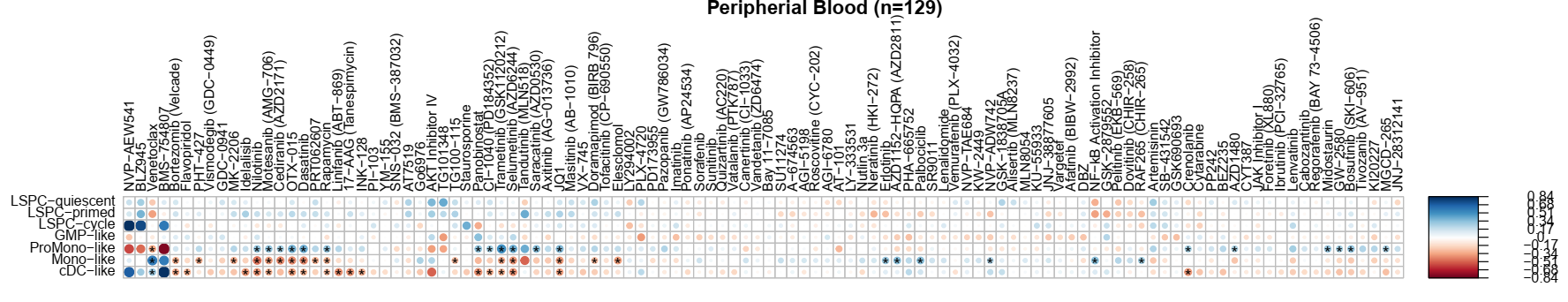


**Figure S4. The correlation plot of 110 drugs containing most significant correlations in group PB**. The annotation is similar to Figure S1.


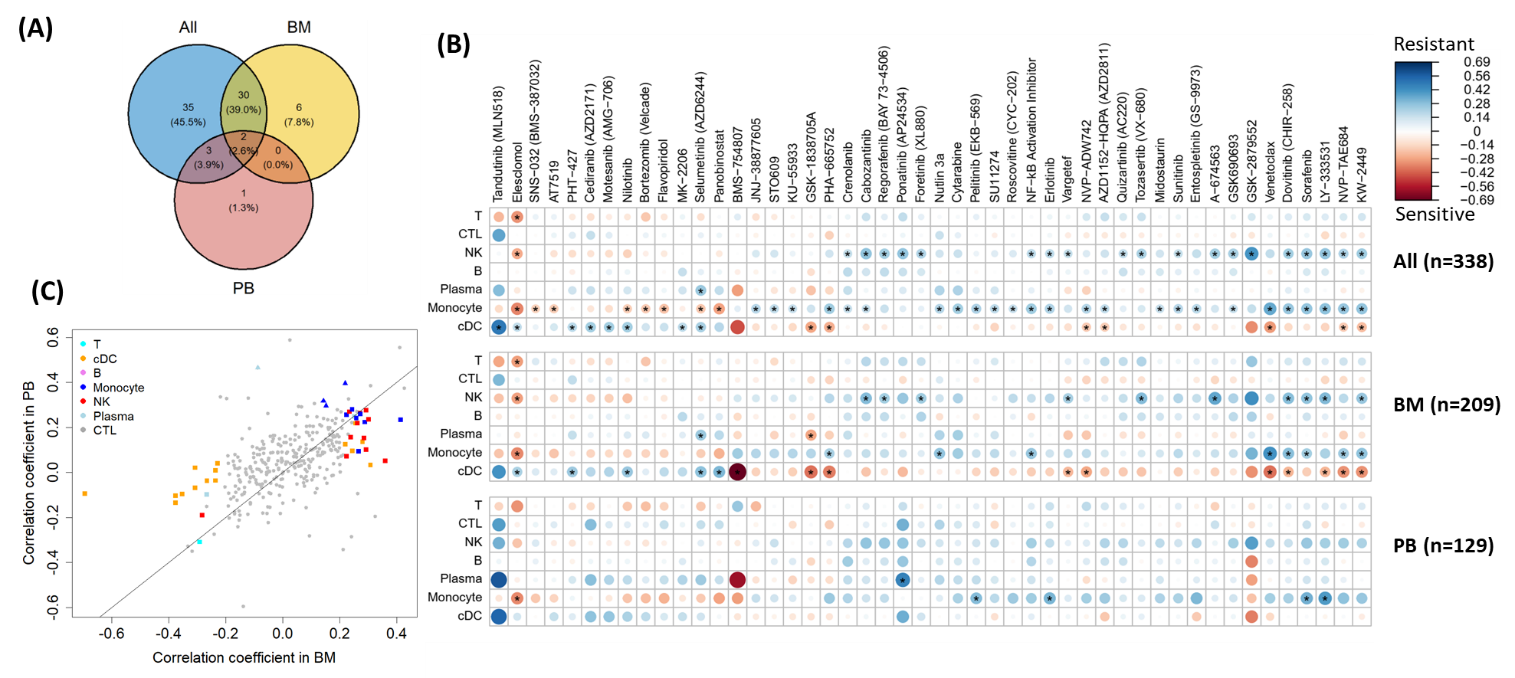


**Figure S5: Association between immune cell types and drug response**. (A) The venn diagram of the significant correlations (FDR < 0.05) between cell-type proportions and drug response across all AML patients (All group), patients with BM samples and patients with PB samples; (B). The correlation plot of all drugs containing most significant correlations across all patient groups. The color and size represent the direction and magnitude of the correlation, respectively. The asterisks indicate the significant correlation (FDR < 0.05); (C) The Spearman correlation coefficients in BM samples vs BP samples.


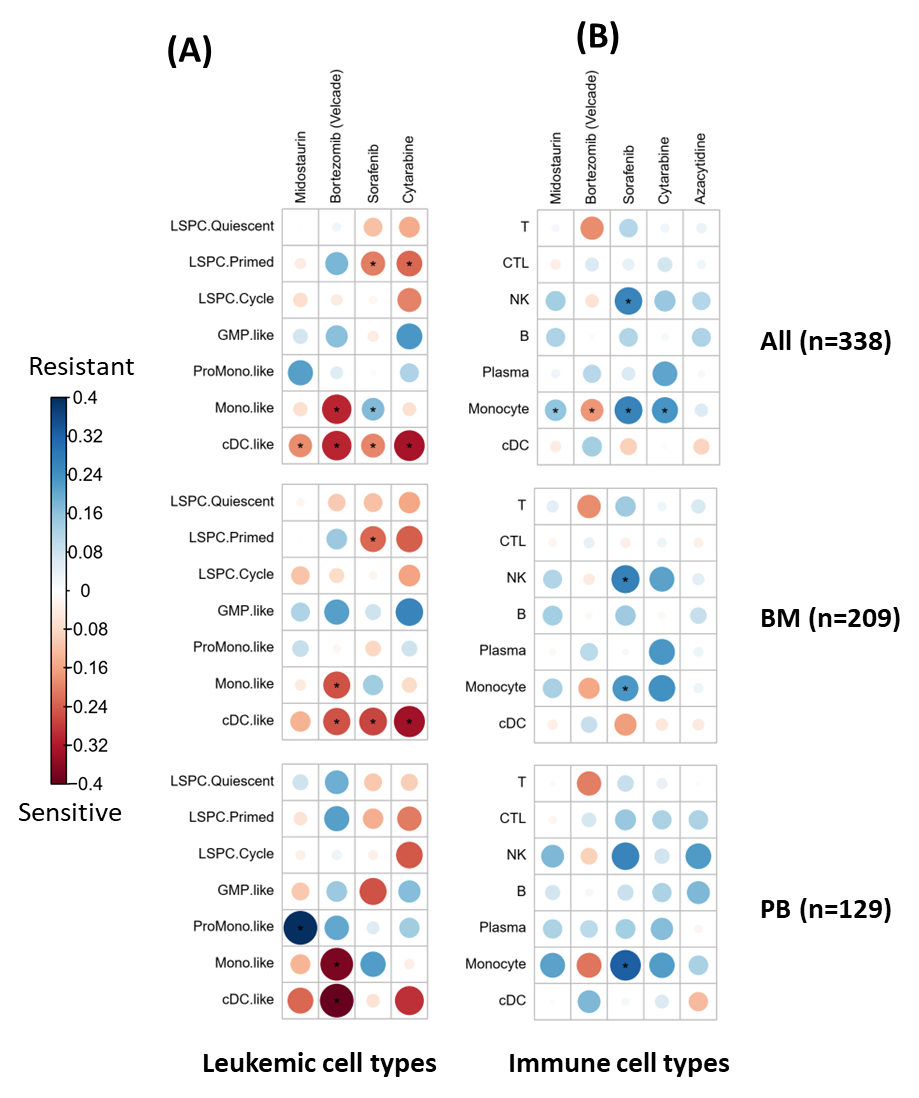


**Figure S6: Association between cell types and drug response from four first treatment drugs with ex vivo drug sensitivity data.** Only drugs with at least one significant correlation (FDR < 0.05) are reported.

**TABLES**

**Table S1.** Comparisons between cell type proportions and specimen types in BEAT-AML cohort. The cell types with bold numbers in the last column are significant with FDR < 0.05.

|  | **Cell Types** | **Bone Marrow Aspirate (N=274)** | **Peripheral Blood**  **(N=169)** | **P Value** | **FDR** |
| --- | --- | --- | --- | --- | --- |
| **Leukemic Cell Types** | LSPC-quiescent | 0.04(0-0.36) | 0.07(0-0.43) | 5.30E-04 | **2.12E-03** |
|  | LSPC-primed | 0.1(0-0.48) | 0.12(0-0.5) | 1.53E-01 | 3.05E-01 |
|  | LSPC-cycle | 0.04(0-0.52) | 0.02(0-0.42) | 1.43E-04 | **7.17E-04** |
|  | GMP-like | 0.17(0-0.74) | 0.05(0-0.61) | 3.12E-08 | **2.18E-07** |
|  | ProMono-like | 0.04(0-0.42) | 0.02(0-0.37) | 4.32E-06 | **2.59E-05** |
|  | Mono-like | 0.03(0-0.5) | 0.02(0-0.45) | 6.38E-01 | 6.38E-01 |
|  | cDC-like | 0.12(0-0.55) | 0.15(0-0.5) | 3.55E-02 | 1.07E-01 |
| **Immune Cell Types** | T | 0.02(0-0.4) | 0.02(0-0.37) | 5.50E-03 | **3.30E-02** |
|  | CTL | 0.01(0-0.09) | 0.02(0-0.13) | 1.19E-03 | **8.34E-03** |
|  | NK | 0.02(0-0.09) | 0.02(0-0.23) | 1.57E-02 | **7.84E-02** |
|  | B | 0.02(0-0.12) | 0.01(0-0.13) | 5.54E-01 | 5.54E-01 |
|  | Plasma | 0.01(0-0.04) | 0.01(0-0.09) | 2.47E-01 | 4.93E-01 |
|  | Monocyte | 0.05(0-0.47) | 0.06(0-0.45) | 7.75E-02 | 2.36E-01 |
|  | cDC | 0.06(0-0.29) | 0.07(0-0.27) | 5.90E-02 | 2.36E-01 |

**Table S2.** List of 40 inhibitors used during first treatment.

| **Drug** | **Frequency** |
| --- | --- |
| Cytarabine | 197 |
| Idarubicin | 141 |
| Fludarabine | 55 |
| Azacitidine | 32 |
| Daunorubicin | 28 |
| Melphalan | 27 |
| Busulfan | 17 |
| ATRA (Tretinoin) | 16 |
| Sorafenib | 15 |
| Arsenic Trioxide | 13 |
| Mitoxantrone | 13 |
| Etoposide | 13 |
| Cyclophosphamide | 10 |
| Filgrastim | 10 |
| Venetoclax (ABT-199) | 10 |
| ATG | 8 |
| TBI | 8 |
| Midostaurin | 8 |
| Decitabine | 6 |
| Ruxolitinib | 6 |
| BI836858 (anti-CD33) | 4 |
| Ivosidenib | 3 |
| Gilteritinib (ASP2215) | 3 |
| Lenalidomide | 2 |
| Enasidenib | 2 |
| Cladribine | 2 |
| Trametinib | 1 |
| Hydroxyurea | 1 |
| Dasatinib | 1 |
| Methotrexate | 1 |
| Entospletinib | 1 |
| SY-1425 (Tamibarotene) | 1 |
| MLN4924 | 1 |
| Clofarabine | 1 |
| Mercaptopurine | 1 |
| BCNU | 1 |
| Idasanutlin | 1 |
| MGB453 | 1 |
| Bortezomib | 1 |
| Thiotepa | 1 |

**Table S3.** Distribution of the number of inhibitors used during the first treatment.

| **How many inhibitors used in first treatment** | **Number of patients** |
| --- | --- |
| 1 inhibitor | 55 |
| 2 inhibitors | 219 |
| 3 inhibitors | 26 |
| 4 inhibitors | 8 |
| 5 inhibitors | 11 |
| 6 inhibitors | 4 |

**Table S4.** Association between cell types and induction therapy. The cell types with bold text are significant with FDR < 0.05.

| **CellType** | **Pvalue** | **FDR** |
| --- | --- | --- |
| **LSPC-Quiescent** | **0.001200** | **0.008403** |
| **LSPC-Primed** | **0.000518** | **0.007252** |
| **LSPC-Cycle** | **0.002496** | **0.01165** |
| **GMP-like** | **0.003758** | **0.013154** |
| ProMono-like | 0.406157 | 0.627512 |
| Mono-like | 0.057389 | 0.133907 |
| cDC-like | 0.035452 | 0.099267 |
| T | 0.879699 | 0.947368 |
| CTL | 0.954815 | 0.954815 |
| NK | 0.837712 | 0.947368 |
| B | 0.706769 | 0.899524 |
| Plasma | 0.442226 | 0.627512 |
| Monocyte | 0.448223 | 0.627512 |
| cDC | 0.446884 | 0.627512 |

**Table S5.** Impact of interactions between cell types and Cytarabine on induction therapy. The cell type with bold text is significant with FDR < 0.05.

| **CellType** | **Pvalue** | **FDR** |
| --- | --- | --- |
| LSPC-Quiescent | 0.977780 | 0.977780 |
| LSPC-Primed | 0.324231 | 0.648462 |
| LSPC-Cycle | 0.626928 | 0.797909 |
| GMP-like | 0.057946 | 0.164869 |
| ProMono-like | 0.613416 | 0.797909 |
| Mono-like | 0.774261 | 0.903305 |
| cDC-like | 0.545898 | 0.797909 |
| T | 0.523554 | 0.797909 |
| CTL | 0.058882 | 0.164869 |
| NK | 0.930352 | 0.977780 |
| B | 0.032199 | 0.150262 |
| Plasma | 0.122462 | 0.285744 |
| Monocyte | 0.024527 | 0.150262 |
| **cDC** | **0.003377** | **0.047284** |

**Table S6.**  Refractory rate after AML induction therapy by cDC level for the **All** group. Low level refers to less and/or equal median of related cell-type proportion whereas high level refers to greater than median.

| **Cytarabine** | **cDC** | |
| --- | --- | --- |
|  | **Low Level (n=162)** | **High Level (n=161)** |
| **Not used (n=126)** | 0.15 (9/60) | 0.38 (25/66) |
| **Used (n=197)** | 0.37 (38/102) | 0.26 (25/95) |

**Table S7.** Refractory ratio after AML induction therapy by cDC level for the **BM** group. Low level refers to less and/or equal median of related cell-type proportion whereas high level refers to greater than median.

| **Cytarabine** | **cDC** | |
| --- | --- | --- |
|  | **Low Level (n=104)** | **High Level (n=103)** |
| **Not used (n=79)** | 0.10 (4/41) | 0.39 (15/38) |
| **Use (n=128)** | 0.37 (23/63) | 0.26 (17/65) |

**Table S8.** Refractory ratio after AML induction therapy by cDC level for the **PB** group. Low level refers to less and/or equal median of related cell-type proportion whereas high level refers to greater than median.

| **Cytarabine** | **cDC** | |
| --- | --- | --- |
|  | **Low Level (n=58)** | **High Level (n=58)** |
| **Not used (n=47)** | 0.32 (6/19) | 0.32 (9/28) |
| **Used (n=69)** | 0.41 (16/39) | 0.23 (7/30) |
